# Diversity of cave Phlebotomines (Diptera: Psychodidae) from a Colombian cave

**DOI:** 10.1016/j.actatropica.2022.106515

**Published:** 2022-05-20

**Authors:** Manuela Velásquez Londoño, Adam M.M. Stuckert, Rafael J. Vivero, Daniel R. Matute

**Affiliations:** a Programa de Estudio y Control de Enfermedades Tropicales, Universidad de Antioquia; b Biology Department, University of North Carolina, 250 Bell Tower Drive, Genome Sciences Building, Chapel Hill, NC 27510, United States; c Grupo de Microbiodiversidad y Bioprospección, Universidad Nacional de Colombia, Sede Medellín

**Keywords:** Sandflies, Leishmaniasis, Vector biology, Cave biodiversity, Species richness

## Abstract

Sandflies are vector species of *Leishmania*, among many other pathogens, with a global distribution and a variety of ecological niches. Previous samplings have found that karstic formations (i.e., caves, grottos, and folds formed by the erosion of limestone) serve as a natural habitat to sandfly species. The majority of samplings of cave sandfly diversity have occurred in Brazil and to date none have studied the species composition in a cave in the Northern Andes. We collected sandflies in the Cave “Los Guácharos”, in the state of Antioquia, Colombia. The sampling was carried out during two consecutive nights in September 2019. CDC-type light traps were installed inside the cavern and in other surrounding karst systems (caves, rock-breaks, and folds). In total, we identified 17 species of sandfly from the cave and surrounding karst systems, including a new record for Colombia (*Bichromomyia olmeca*), and provide the first karstic reports for four other species (*Lutzomyia gomezi, Lutzomyia hartmanni, Pintomyia ovallesi*, and *Psychodopygus panamensis*). We then used the results of our survey and published literature to test two hypotheses. First, that sandfly diversity in Neotropical caves is richest nearer to the equator, and second that there is a phylogenetic signal of karstic habitat use in sandflies. Counter to our predictions, we found no evidence that diversity follows a latitudinal gradient. Further, we find no evidence of a phylogenetic signal of karstic habitat use, instead finding that the use of caves likely evolved multiple times across several genera. Our results highlight the importance of a wide sampling to understand the natural habitat of sandflies and other disease vectors.

## 1. Introduction

The family Psychodidae is a speciose group of dipterans that is globally distributed. The family encompasses seven different subfamilies and has over 4,000 species (Pave et al., 2011; Bejarano and Estrada 2016). The majority of the species diversity is encompassed by two subfamilies. The subfamily Psychodinae (Wagner 2004; Espíndola et al., 2012) commonly referred to as moth flies or drain flies, which includes 2,000 species, many of which are human commensals (Cordeiro and Wagner 2018; Munstermann 2019). The second subfamily, Phlebotominae, includes 500 species, of which over 90 are vectors of diseases of humans and other animals alike (Killick-Kendrick 1990; Ready 2013). These species are commonly referred to as sandflies and are the main vectors of Leishmaniasis in the tropics and subtropics. Just for Leishmaniasis, one of the diseases transmitted by members of the subfamily Phlebotominae, more than 12 million people are infected and over 2 million new cases are reported annually. The number of recorded deaths due to *Leishmania* infection per year is around 60,000, a number that is, in all likelihood, a vast underestimate due to inadequate reporting requirements and *Leishmania* prevalence in areas with minimal access to healthcare (Alvar et al., 2012; Okwor and Uzonna 2016; Karimkhani et al., 2016; Bailey et al., 2017). Sandflies also transmit other pathogens that cause severe diseases, among which are three arboviruses (Sicilian virus, Naples virus, and Toscana virus; (Tesh et al., 1975; Batieha et al., 2000; Dionisio et al., 2003; Izri et al., 2008)), *Vesiculovirus* (Comer and Tesh 1991), *Orbivirus* (Depaquit et al., 2010; Phumee et al., 2021), *Flavivirus* (Acevedo et al., 2008), and *Bartonella bacilliformis*, the etiological agent of Carrion’s disease (Caceres et al., 1997).

Species from the Psychodidae family occupy a variety of habitats. Psychodinae species are often associated with synanthropic habitats but are also commonly found in drains and aquatic environments (Cordeiro and Wagner 2018). Phlebotominae sandflies are often associated with places where they can obtain blood meals such as human settlements (Ramos et al., 2014) and coffee plantations (Ferro et al., 2015). Mating often occurs at lekking sites at the base of large trees (Memmott 1991, 1992). Females tend to oviposit close to these lekking sites in clay rich soils. Painstaking collections have revealed the presence of immature stages in such sites (Rutledge and Mosser 1972; Vivero et al., 2015). The specificity of habitat varies across species, and in some cases even populations, and while some species are geographically widespread and inhabit a variety of habitats others seem to be more restricted in their geographic and ecological distribution. The precise details of habitat usage by most species remain unknown (Alexander 2000).

One of the potential ecological niches of sandflies is karstic landscapes (Oca-Aguilar et al., 2013; Blavier et al., 2019; Costa et al., 2021). Karstic landscapes are irregular limestone formations affected by erosion that encompass fissures, sinkholes, underground streams, and caverns. Previous reports have identified that some Phlebotomine species frequently breed in cavern systems. Quate (1962) carried out the first cave collection in the Batu Caves (Malaysia) and identified 22 phlebotomids from different subfamilies. Since then, it has become clear that cavern soil is a potential breeding ground for Psychodids, especially for Phlebotomines (e.g., Quate 1962; Galati et al., 2003; Alves et al., 2011; Campos et al., 2017; Costa et al., 2021). Several species are exclusively found in caves and breeding in caves is a trait that is widespread in the family (Alves et al. 2008; Carvalho et al. 2010; Carvalho et al., 2011; Barata et al., 2012; Vilela et al., 2015). Cave surveys have the potential to add crucial information about the taxonomic and ecological diversity of sandflies.

South and Central America harbor a high diversity of Phlebotomine species (D’Agostino et al., 2022) and they house the vector genus *Lutzomyia sensu lato*. Samplings from Brazil suggest the existence of species restricted to caves (Alves et al., 2011; Carvalho et al., 2013; Campos et al., 2017), and that important disease vectors can breed in caves (Cutolo et al., 2008; Carvalho et al., 2017). Few efforts have addressed the diversity of this group in caves in the Neotropics, especially in Northern South America. The only study on this matter found Phlebotomines associated with a karstic, not cavernous, system in the Cañón del Río Claro Natural reserve (Antioquia, Colombia). Bejarano et al. (2018) reported the presence of *Warileya (Hertigia) hertigi*, a Phlebotomine not involved in the transmission of disease. Addressing the diversity of Phlebotomines in caves in the Northern Andes holds the potential to reveal general patterns about the diversity of this vector group, which remains a largely unaddressed question.

We expanded the previous sampling of Bejarano et al. (2018) and collected Phlebotomines in the cave system in the Los Guácharos Cave in the Cañón del Río Claro Natural reserve (Antioquia, Colombia). Our point sampling revealed a species previously unknown to occur in Colombia, and four species that were not known to occur in karstic environments. We then used our findings as well as existing datasets to test the hypothesis that Neotropical cave sandfly species diversity follows the expectations of a latitudinal species richness gradient. We find that cave sandfly species diversity does not follow the expectations of a latitudinal species richness gradient and that the species diversity in tropics and subtropics seems to be similar. Finally, we used comparative phylogenetic methods to study whether association to karstic environments has evolved multiple times in the evolutionary history of the Psychodidae family. We find that association to karst is not a trait with a strong phylogenetic signal. Our findings suggest that the ecological and evolutionary implications of cave diversity is fundamental for the understanding of species richness in the family.

## 2. Methods

### 2.1. Locality

The focus of our collection was the cave system at the Cañón del Río Claro Nature Reserve. The reserve is located between the municipalities of Sonsón, Puerto Triunfo and San Luis, to the southwest of the department of Antioquia (5° 5 ’N, 74° 39′ W, (Vivero et al., 2010). This cave system is of karstic origin and was formed by the erosion of irregular limestone (Restrepo Martinez 2011). The largest cave of the cave system, the Los Guácharos cave, has a length of 442.8 m, with an entrance 10 to 15 m high by 2 to 3 m wide and is considered a humid cave (Moncada et al., 1989). The environment inside the karstic formation can be classified as caverns, fissures, sinkholes, and underground streams. The annual mean temperature and mean relative humidity of the cave are 22 °C and 80% respectively. The cave serves as a roosting site for oilbirds (*Steatornis caripensis*) and chestnut long-tongued bats (*Lionycteris spurrelli*, Caballero et al. 2021).

### 2.2. Sampling

**Fig. 1.**
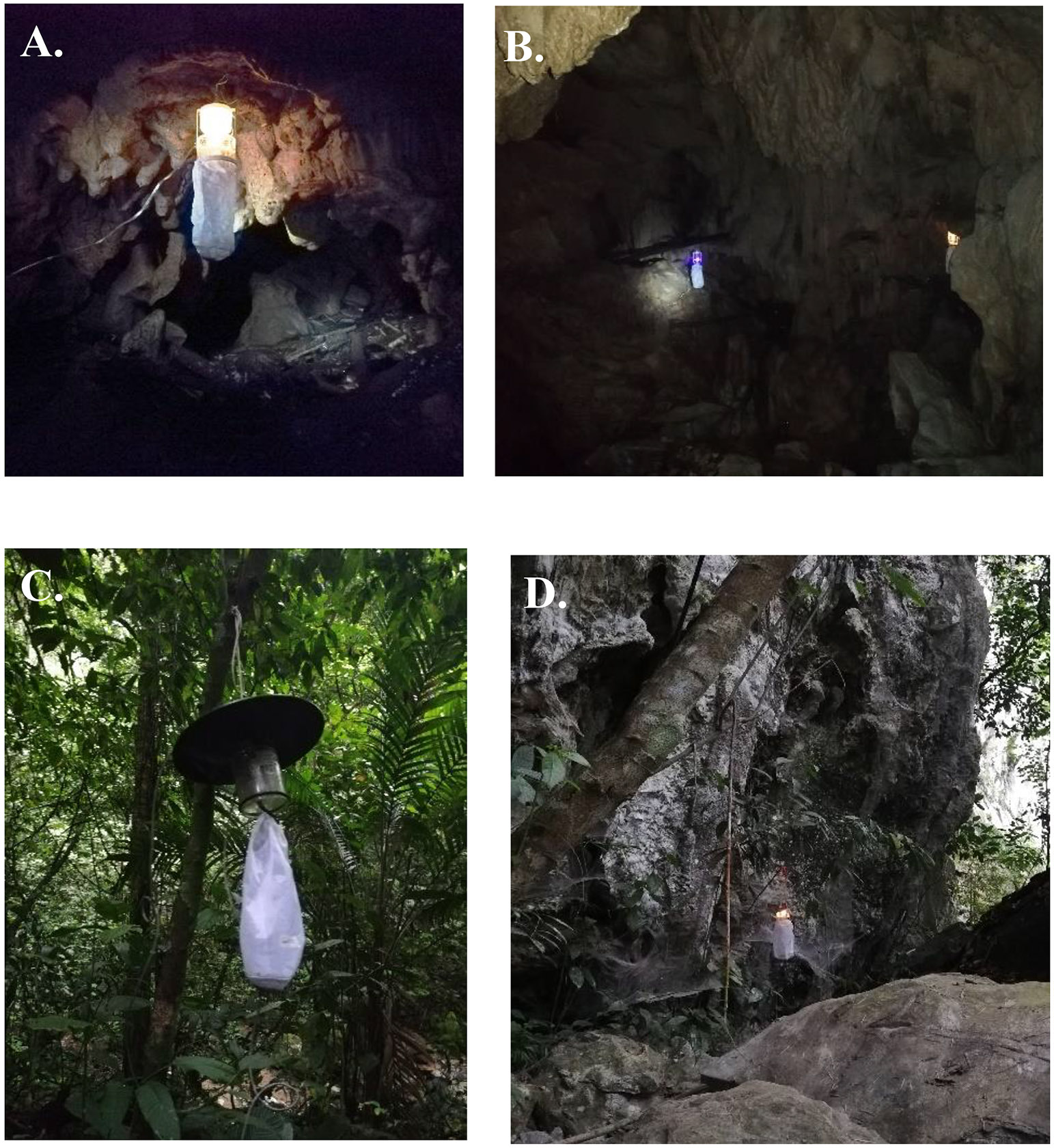
CDC light traps in the Los Guácharos cave collection. **A-B.** Cave. **C.** Vegetation outside the cave. **D.** Karstic folds.

To characterize the entomofauna in the cave, we carried out a sampling during two consecutive nights in the month of September 2019. We installed 10 CDC-type light traps for a period of 12 h starting at 18:00. The traps were collected at 6:00 the following morning. To attract blood feeding insects, we placed dry ice under the traps. The sublimating CO_2_ mimics the respiration of vertebrates and increases the sandfly yield. Two of the CDC traps were located in a cave with a 2 m^2^ cavity. Two more traps were placed inside a karst fold with a depth of 4 m and an altitude of approximately 4 m. The other six traps were placed in the main gallery of the cavern. The location of the traps obeyed safety precautions and the depth of bodies of water (Fig. 1). For comparison, we installed a CDC trap in the vegetation outside the cave. We compared the species richness outside and inside the cave.

For species identification, we transported the nets of the CDC traps to the Laboratory of Medical Entomology of the Program for the Study and Control of Tropical Diseases of the University of Antioquia. We extracted specimens from the traps using an aspirator and placed them in a Petri dish and inspected under a Leica microscope. To clear samples, we immersed each sandfly in lactophenol (1:1 ratio). Finally, we identified samples to species-level following sandfly taxonomic classification keys (Galati 2019, Young and Duncan 1994, Ibañez-Bernal 1999).

### 2.3. Species diversity and comparison to other studies

Next, we calculated metrics of species diversity in the Los Guácharos cave and compared them to other cave collections in the Neotropics to determine whether sandfly species richness followed a latitudinal gradient. We only included studies for which the raw data was reported (i.e., all the species collected during the sampling and their respective abundances), we compiled data from 32 other cave samplings. Using this dataset, we calculated three different metrics of species diversity. First, we used the number of collected species (i.e., species richness). Second, we used the Shannon’s Index (H) which is a weighted geometric mean of species abundances. H follows the form:

$$H=\sum_{i=1}^{R}[p_{i}\times ln(p_{i})]$$

where $p_{i}$ is the proportional representation of a given species and R is the number of species in the sample. If most of the abundance in the sample is concentrated in a single species, then H will be 0. In cases in which all the species are equally abundant, H will equal ln(R).

We calculated a third index of diversity, species evenness, which is defined as H normalized by the number of species and follows the form:

J=H∕log(species number)

J ranges from 0 (i.e., low evenness, presence of a dominant species) and 1 (i.e., complete evenness, all species represented equally. All metrics were calculated using the R package *vegan* (function *diversity*, (Oksanen 2013; Oksanen et al., 2013, 2015)).

Different samplings had different levels of effort. To account for differences in sampling effort, we used a rarefaction approach in which we estimated the expected number of species in each locale with the smallest number of collected samples from a published cave sampling study which we have included in our expanded dataset (*N* = 6). We also estimated rarefaction curves for all the 33 localities. For these analyses we used the functions *rarefy* in the R package *vegan* and used 1000 bootstrap replicates per locality (Oksanen 2013; Oksanen et al., 2013, 2015). This function calculates a rarefaction curve and estimates total species abundance for a given sample size (in our case *N* = 6), thus correcting for sampling effort across studies.

We next studied whether there was a relationship between different metrics of cave sandfly species richness (both before and after rarefaction) and latitude. Our expectation was that species richness in caves would follow a latitudinal pattern of diversity and that caves closer to the tropics would show higher species richness, as has been observed for other taxa (Mittelbach et al., 2007; Mannion et al., 2014; Jablonski et al., 2017; Pontarp et al., 2019). We used linear regressions to assess whether the different metrics of species richness were associated with the latitude of the cave (function *lm*, library *stats*; R Core Team 2021). We bootstrapped the regression coefficients using the function *Boot* (*R* = 1,000; library *car*, Fox et al., 2012. Fox and Weisberg 2019).

### 2.4. The evolution of cavernicolous niche

Our work and others report several unrelated species in the Pscychodidae family to have a cavernicolous niche. This suggests the possibility that the niche has evolved several times in the evolutionary history of the group. We formally addressed whether cavernicolous niche has evolved more than once in the evolutionary history of the family using comparative phylogenetic methods. We used a previously published phylogeny of the group using four different loci (D’Agostino et al., 2022) and classified each species as cavernicolous (i.e., has been collected in caves) or non-cavernicolous. We measured whether cave habitat followed a phylogenetic pattern. We used Pagel’s *λ* (Pagel 1999) for discrete traits (i.e., whether a species had been found in caves or not). Pagel’s *λ*, is a measure of phylogenetic signal which estimates the extent to which the phylogenetic history of a clade is predictive of the trait distribution at the tree tips. Values of *λ* lower than 1.0 represent traits being less similar amongst species than expected from their phylogenetic relationships. A *λ* equal to 1.0 suggests that traits covary with phylogeny (Pagel 1997, 1999) and is consistent with niche conservatism along the phylogeny (Cooper et al., 2010). A value close to zero indicates no effect of the phylogenetic history on the evolution of a trait. We used the wrapper *fitLambda* for the *ace* function (library *ape*, Paradis et al., 2004; Paradis and Schliep 2019) for ancestral character estimation and *λ* calculation. This function optimizes Pagel’s *λ* tree transformation for a discrete character evolving by a continuous-time Markov chain. We also used the function *fitdiscrete* (library *geiger*, (Harmon et al., 2008; Pennell et al., 2014)) which yielded the same results.

## 3. Results

### 3.1. Collection description

We collected 52 sandflies, 16 males and 36 females. This difference in the sex ratio of the collection obeys different patterns of attraction to light traps (Toprak and Özer 2007). The samples belonged to 17 species of the Phlebotominae subfamily, representatives of the genera *Warileya, Lutzomyia, Micropygomyia, Bichromomyia, Evandromyia, Pintomyia, Pressatia, Psathyromyia, Pschoathyrdoomyia, Pschoathyromygusia*, and *Helcorcitomyia* (Table 1). The most abundant species was *Warileya* (*Hertigia*) *hertigi* (21.14%), followed by *Pintomyia ovallesi* (17.3% of total samples), and *Lutzomyia gomezi* (11.53%; Table 1). This collection represents two novel findings. First, one of the collected species (*Bichromomyia olmeca nociva*) is novel for Colombia which significantly expands the range of the species. Second, we report four species that had not been, or have been rarely, collected in caves.

### 3.2. New report of Bichromomyia olmeca nociva for Colombia

Our sampling includes one species that had not been reported for Colombia. We describe the morphological features of this specimen as follows.

#### 3.2.1. Bichromomyia olmeca nociva

**Fig. 2.**
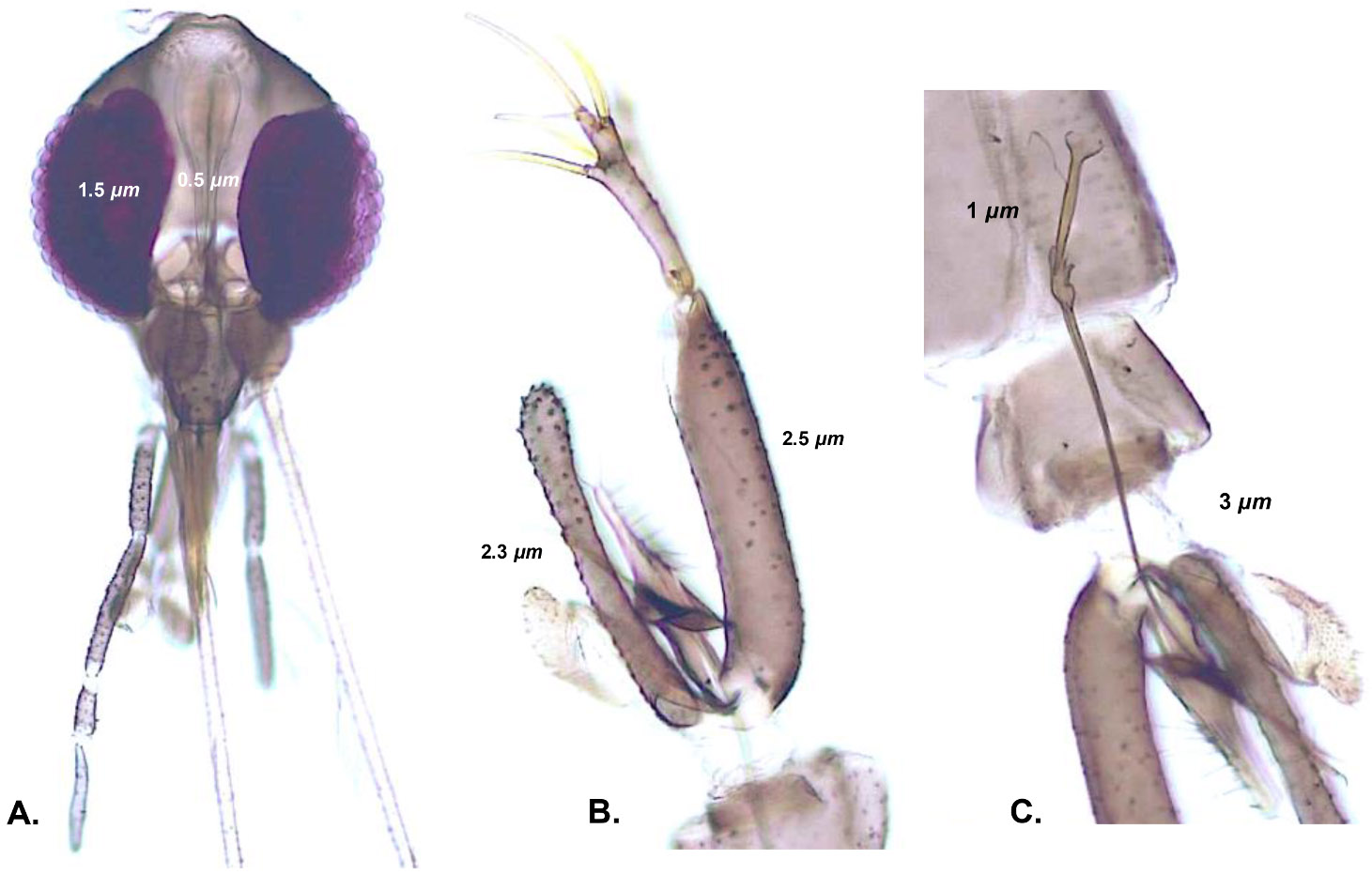
Diagnostic traits of the new Phlebotomine register for the Colombian territory. *Bichromomyia olmeca nociva:* (a) Head and interocular space, (b) Epandrial lobes slightly shorter than gonocoxite, (c) spermiducts/sperm pump.

(Young and Arias 1982): folding (5° 53′ 16.8729 ”N, 74 ° 51′ 17.6029″ W), Cañón del Río Claro Nature Reserve (Antioquia); Fig. 2A-C. (♂), interocular distance (1.5 mm) equivalent to 1/3 of the width of the eye (0.5 mm), epandrial lobe (2.3 mm) shorter than the gonocoxite (2.5 mm), ratio of aedegal ducts (3.0 mm) / sperm pump (1.3 mm) equals to 3.0:1.0. *Bichromomyia olmeca nociva* was found in the inner part of the cave across the river. Before this report, *B. olmeca nociva* has been reported for the Brazilian Amazon but not for the Colombian territory (~1,700 km extension).

### 3.3. New reports for cave-dwelling species

Our sampling found four species that have not been collected in caves, and one species which has only been reported in a cave once before. In this sampling, we found that *Lutzomyia hartmanni* (Fairchild and Hertig 1957) appeared associated with two different karstic environments, caves and rock-breaks. *Lutzomyia gomezi* (Nitzulescu 1931) has not been reported before in association with caves or any karstic systems. However, in this report we find the species in the Los Guácharos cave and surrounding vegetation. In both instances we found females that recently had a blood meal. Finally we observed two more species, *Psychodopygus panamensis* (Shannon 1926) and *Pintomyia ovallesi* (Ortiz 1951) in caves for the first time in our sampling. *Psychodopygus panamensis* is a generalist species that is often characterized as anthropophilic (González et al., 1999; Santamaría et al., 2020; Rigg et al., 2021) as is *Pintomyia ovallesi* (Feliciangeli 1997; Feliciangeli and Rabinovich 1998; Rabinovich and Feliciangeli 2004). One previous study has collected samples from this species in a cave in Belize (Williams 1976). In both the Belize collection and our collection, females of *Psychodopygus panamensis* had blood-fed. Finally, we found *Bichromomyia flaviscutelata* (Mangabeira Filho 1942) in the Los Guácharos cave, agreeing with a previous report in Brazilian caves that reported this species can have a cavernicolous habitat (Alves et al. 2011).

### 3.4. Species richness and comparisons to other caves

Our sampling yielded 52 individuals and revealed the existence of 17 species in the Los Guácharos cave. This sample has one of the highest species richness in any Neotropical sample collected in caves (Table 2). We also calculated the Shannon diversity index for each location and a normalized evenness index for 32 caves sampled in the Neotropics. The Guacharos cave also has the highest Shannon’s Index (H) and the one of the highest evenness indexes of any Neotropical cave (Table 2) suggesting one of the highest diversity of sandflies in Neotropical caves. Nonetheless, our sample has a relatively low sample size compared to others. To account for differences in the sample size, we rarefied two of the diversity indexes and the results are qualitatively similar to those before rarefaction (Table 2). Regardless of the species metric diversity, the Los Guacharos cave has a high species diversity compared to other samples.

**Fig. 3.**
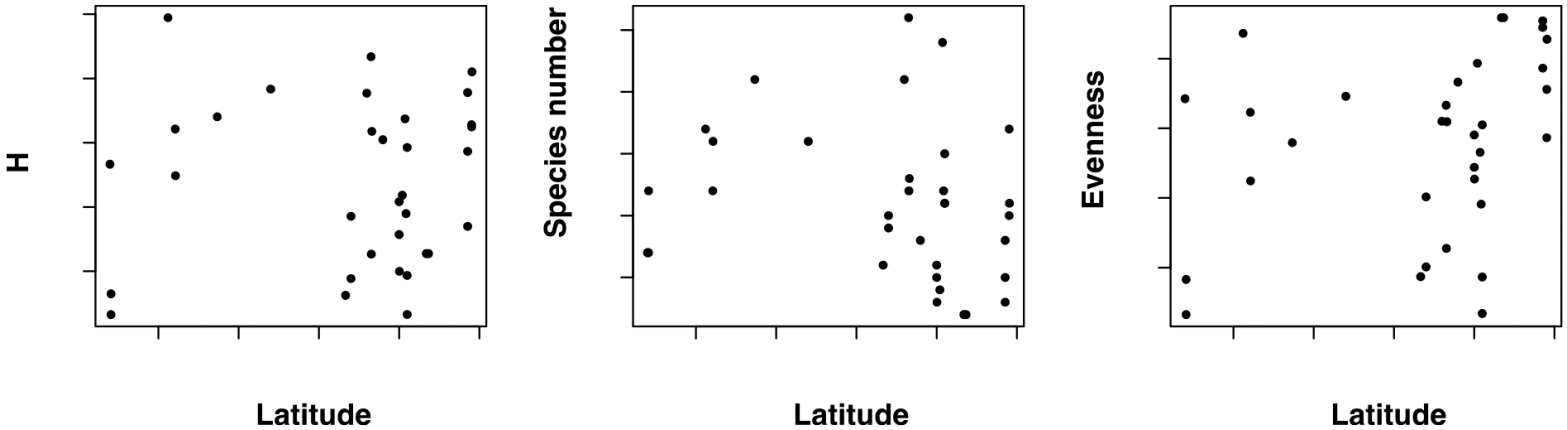
Three different metrics of the species diversity in Neotropical sandfly cave collections. **Left.** Shannon’s H index **Center**. Number of species **Right**. Evenness. Each point represents the species richness metric for a sampling listed in Table 2. Significance of the regressions is shown in Table 3. None of the three metrics shows evidence for a latitudinal gradient of sandfly species diversity collected in caves.

Next, we tested whether the diversity of sandflies in caves followed a latitudinal gradient of diversity by regressing the latitude of the collection with each of the three metrics of diversity (Fig. 3). Contrary to our expectation, we find that none of the regressions is significant suggesting that species diversity of sandfly caves does not decrease as the cave gets farther from the Equator. The result was the same with any of the three diversity indexes regardless of whether we corrected for effort or not (Table 3).

### 3.5. Phylogenetic signal of cavernicolous habitat

**Fig. 4.**
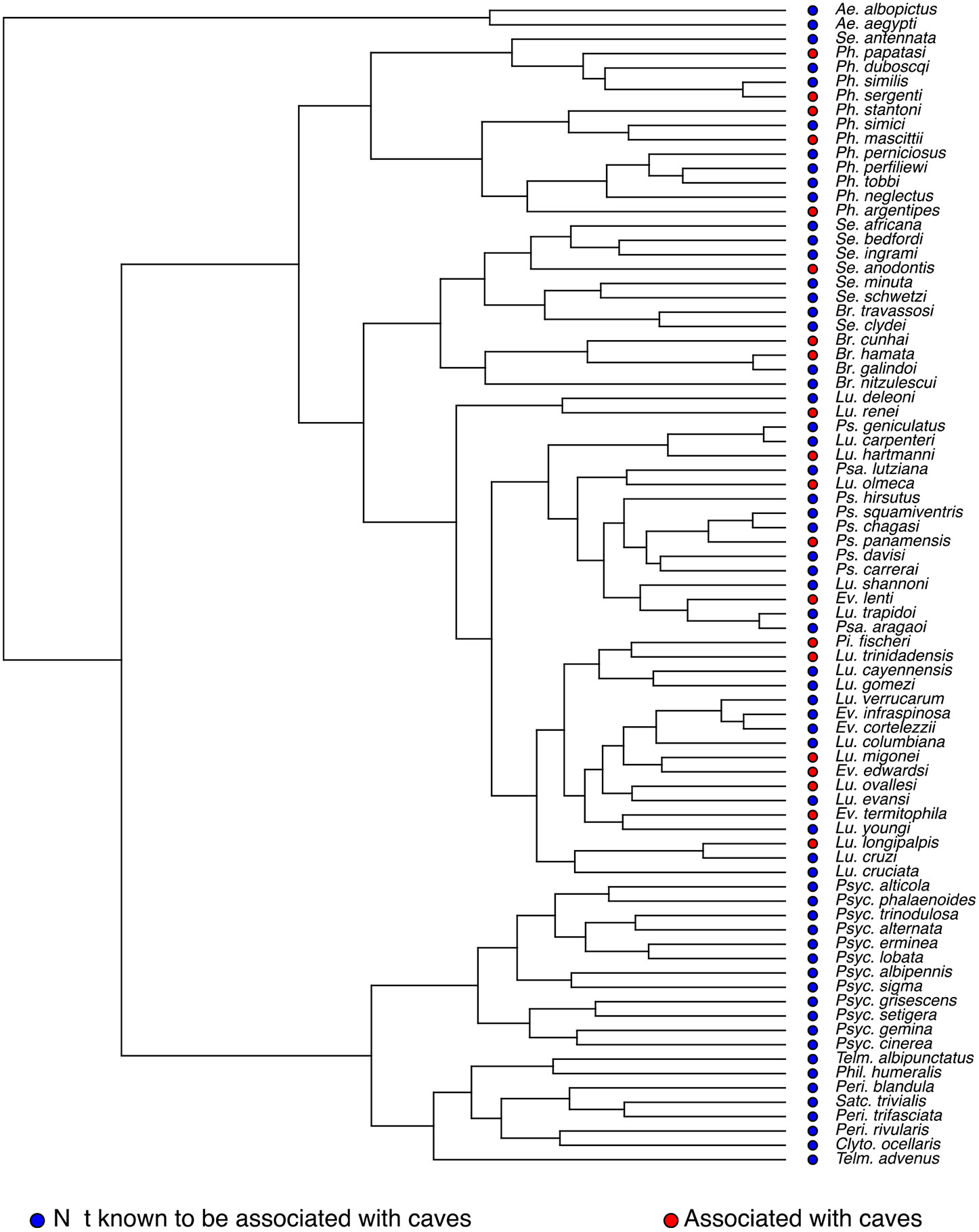
Phylogenetic signal of cavernicolous habitat in Psychodidae. The dots at the tree tips show the extant known habitat choice for each species in the phylogeny. *Ae: Aedes; Se: Sergentomyia*; pH: *Phlebotomus*; *Br: Brumptomyia; Lu: Lutzomyia; Ps: Psychodopygus; Psa: Psathromyia; Ev: Evandromyia; Pi: Pintomyia; Psyc: Psychoda; Telm: Telmatoscopus; Phil: Philosepedon; Satc: Satchelliella; Peri: Pericoma; Clyto: Clytocerus*. Blue: not known to be associated with caves, Red: associated with caves.

We used the Psychodidae phylogenetic tree to study whether the cavern habitat association had a phylogenetic signal in the family. Our metric of phylogenetic signal was close to zero (Pagel’s *λ* = 6.611 × 10^−5^; logLik = −45.273) which suggests no effect of the phylogeny on the distribution of cavernicolous habitat. The distribution of the trait along the tree suggests the same pattern as cavernicolous species are not related to each other (Fig. 4). Thus cave use likely evolved at least three times in the family as it appears in at least three different genera.

## 4. Discussion

The study of the habitat of vector species can yield important information on potential control strategies to limit their ability to transmit disease. We did a point sampling of a cave in western Colombia. Our report is the first multispecies sampling of a cave in the northern Andes. We report that a *Bichromomyia olmeca* has a larger geographic range than previously reported (Shimabukuro et al., 2017; de Melo et al. 2020). The species is naturally infected and can effectively transmit *Leishmania amazonensis* (Brazil et al. 2015), which highlights the importance of finding this species in cave systems relatively close to human settlements and with frequent ecotourism. *Bichromomyia olmeca* is found throughout Central America to Brazil (GBIF: DOI: https://doi.org/10.15468/dl.g77tbs, years: 1953–2018, Table S1 in Moo-Llanes et al., 2013, and Galati 2018) and seems to be a species complex composed of at least four different lineages: *B. o. nociva* (reported in this study), and *B. o. olmeca* (mainly localized in Mexico), *B. o. bicolor* (reported in Pánama, Colombia, and Brazil), and *B. flaviscutellata sensu stricto* (de Melo et al. 2020). The Guacharos cave harbors both *B. olmeca nociva* and *B. flaviscutellata* and adds to the list of locales where lineages of the *B. olmeca* species complex coexist in the same geographic range (Fairchild and Theodor 1971; Escobar Vasco 1989; Romero Ricardo et al. 2013; de Melo et al. 2020; Santatana et al. 2020). The identification of *B. olmeca nociva* in Colombia poses the need to revisit the geographic range of each species, with a special emphasis on areas of coexistence which might facilitate gene flow between vector species.

Determining range limits has practical implications for our understanding of vector biology. Under current global warming scenarios, *B. olmeca* is predicted to expand its current range to subtropical and temperate areas (Moo-Llanes et al., 2013). This prediction was only possible after a systematic habitat characterization of the species habitat (108 collection sites, Table 1 in (Moo-Llanes et al., 2013) which in turn revealed the suitable current and future ecological niche of the species. To date, the habitat characterization of *B. olmeca nociva* or other species in the species complex, —at least to the same detail as *B. o. olmeca*, —is lacking. We hypothesize that lack of systematic sampling in the Northern Andes might explain the discontinuous geographic range of other species.

More generally, we used the data from our sampling to infer ecological and evolutionary patterns about cave habitat use in sandflies. We find that the Los Guacharos cave has the largest species diversity of any sampled Neotropical cave. Opposite to our expectations, this analysis revealed that sandfly cave species diversity does not follow a latitudinal gradient in the South American continent. Latitudinal gradients, in which the tropics are the most diverse locales, have been reported for almost all systematically sampled taxa (reviewed in Mannion et al., 2014; Jablonski et al., 2017; exceptions listed in Kindlmann et al., 2007). Nonetheless, the drivers of the pattern are hotly debated and are likely to be caused by a variety of processes. Fish for example show higher diversity in the tropics (Mittelbach et al., 2007; Stuart-Smith et al., 2013) but a higher speciation rate in temperate areas (Rabosky et al., 2018). Our results find that the variance of species richness might be the largest in the tropics, as the samples close to the equator show one of the highest (Los Guácharos cave, Colombia; this study) and some of the lowest species richness (Gruta dos Animais, Amazonas, Brazil; Alves et al., 2011) of all the sampled caves. More systematic sampling will be needed before the hypothesis that the tropics have a larger variance in species richness across localities, but the possibility is tantalizing.

Finally, we assessed the evolution of cave habitat choice across the family Psychodidae. We find no phylogenetic signal for cave-habitat association, which supports the possibility that habitat association has evolved repeatedly in the family. Other metrics of the ecological niche of sandflies are highly conserved in the sandfly phylogeny. Proxies of climatic niche are more conserved than expected by the phylogeny (D’Agostino et al., 2022), suggesting that niche conservatism exists within the family for certain traits. A different possibility is that many species are generalists that can use caves as a habitat when present, but limited sampling makes verifying this a challenging hypothesis to test. In this scenario, species are only cavernicolous when there are available caves nearby. The current sampling does not allow us to differentiate between these possibilities. Mark recapture experiments releasing sandflies at the entrance of caves and determining their habitat choice should reveal whether the collection of species in caves is due to happenstance. Regardless of the level of niche conservation of cave use in the family, caves seem to be a niche that is used widely in sandflies and their ecological characterization might be of importance for understanding disease dynamics and vector control.

Our study has caveats that are worth pointing out. First, our sampling is not systematic and did not address the variation of diversity along the year. A proper systematic sampling that addresses species diversity seasonally (at least in the dry and wet seasons) will be necessary to understand the true potential of Colombian caves as reservoirs of vector species . The limitation of systematic sampling extends to other studies (but not all; e.g., Carvalho et al., 2013), and has implications to our inferences of the patterns and processes that lead to the phylogenetic signal of cave species. We note that our results remain qualitatively the same after accounting for this lack of systematic sampling by correcting for sampling effort. Despite this, a more expanded sampling of different caves in the Northern Andes is warranted. One additional caveat is that we expect the phylogeny of sandflies to change as data for more species and better genetic markers become available. The current topology is based on four loci and only includes 79 Psychodidae species. In all certainty, years to come will see an increase on the amount of genomic data in sandflies and this will lead to a revision of the phylogenetic relationships between these species, including some that have cavernicolous habitats. Our estimates of phylogenetic signal will thus change slightly. However, our conclusion that cavernicolous habitat has evolved in several instances in the phylogeny is likely to stand unless the topology is dramatically revised. Our results confirm the need for thorough and systematic sampling of sandfly diversity, including habitats that have not often been sampled (e.g., caves), in order to better understand this ecologically and medically important clade.

## Figures and Tables

**Table 1:** List of Phlebotomines collected in the Los Guácharos Cave in the Rio Claro Natural reserve (Antioquia), Colombia. *F*= female; *M*= male. * = species collected in Colombia for the first time. The number within the parentheses shows the number of gravid females or males with rotated genitalia (reproductive stage).

Species	Cave		Rock-breaks		Fold		Vegetation		Total
	F	M	F	M	F	M	F	M	
*Pintomyia ovallesi*	0	0	6 (1)	0	3(2)	0	0	0	9 (17.3%)
*Lutzomyia gomezi*	0	0	1(1)	0	3(3)	0	2(2)	0	6 (11.53%)
*Warileya hertigi*	5	5	1 (1)	0	0	0	0	0	11 (21.14%)
*Micropygomyia trinidadensis*	0	0	2(1)	0	0	0	1	1	4 (7.69%)
*Psathyromyia carpenteri*	0	0	3 (3)	1	0	0	0	0	4 (7.69%)
*Psychodopygus panamensis*	0	1	0	0	1	1	0	0	3 (5.7%)
*Brumptomyia hamata*	0	1	0	1	0	0	0	0	2 (3.84%)
*Evandromyia saulensis*	0	0	0	0	1	0	1	0	2 (3.84%)
*Helcorcitomyia* sp.	1 (1)	0	1	0	0	0	0	0	2 (3.84%)
*Lutzomyia hartmanni*	1 (1)	0	1	0	0	0	0	0	2 (3.84%)
*Bichromomyia flaviscutelata*	0	0	0	0	1 (1)	0	0	0	1 (1.92%)
Bichromomyia olmeca nociva*	0	0	0	0	0	1	0	0	1 (1.92%)
*Lutzomyia sanguinaria*	0	0	0	0	1	0	0	0	1 (1.92%)
*Micropygomyia atroclavata*	0	0	0	1	0	0	0	0	1 (1.92%)
*Micropygomyia micropyga*	0	0	0	0	0	1	0	0	1 (1.92%)
*Pressatia camposi*	0	0	0	0	0	1	0	0	1 (1.92%)
*Warileya* sp.	0	0	0	1	0	0	0	0	1 (1.92%)
Total Juveniles(reproductive)	7(2)	7	15 (7)	4	10(6)	4	4 (2)	1	52 (100%)
**Total (%)**	**14 (26.9%)**		**19 (36.53%)**		**14 (26.90%)**		**5 (9.61%)**		

**Table 2:** Compilation of neotropical studies of sandfly cave diversity and their respective metrics of species diversity. H: Shannon’s H index. J: Evenness. *Includes estimates over multiple years. ^&^includes more than one connected cave.

Locality	Reference	Before rarefaction			After rarefaction(1,000 replicates; 6 species)	
		*H*	Species richness	Evenness	*H*	Evenness
**Los Guácharos Cave, Colombia**	This study	2.474	17	0.873	1.560	0.448
**Monte Cristo, Brazil**	(Barata et al., 2012)	1.589	13	0.619	0.693	0.253
**Salitre, Brazil**	(Barata et al., 2012)	0.633	12	0.255	1.011	0.506
**North Rondônia, Brazil**	(Ogawa et al. 2016)	1.702	21	0.559	1.011	0.443
**South Rondônia, Brazil**	(Ogawa et al. 2016)	1.918	16	0.692	1.330	0.253
**Gruta dos Animais, Brazil**	(Alves et al., 2011)	0.163	12	0.065	0.000	0.434
**Gruta do Maruaga, Brazil**	(Alves et al., 2011)	0.324	7	0.166	0.000	0.225
**Gruta dos Lages, Brazil**	(Alves et al., 2011)	1.333	7	0.685	0.451	0.366
**Millonario, Belize** *	(Williams 1976)	0.312	6	0.174	0.451	0.000
**Augustine, Belize**	(Williams 1976)	0.443	9	0.201	0.637	0.000
**SA, Belize**	(Williams 1976)	0.928	10	0.403	0.451	0.347
**Bodoquera, Brazil**	(Galati et al., 2003)	0.949	12	0.382	1.011	0.434
**Pitangueiras, Brazil**	(Galati et al., 2003)	0.163	11	0.068	0.000	0.225
**Anhumas, Brazil**	(Galati et al., 2003)	0.467	15	0.173	0.000	0.225
**Guaicurus, Brazil**	(Galati et al., 2003)	1.463	11	0.610	1.330	0.896
**Jardim, Brazil**	(Galati et al., 2003)	0.637	2	0.918	0.636	0.318
**Yucatan, Mexico**	(Oca-Aguilar et al., 2013)	1.041	6	0.581	0.868	0.506
**Ibitipoca, Brazil**	(Carvalho et al., 2011)	0.637	2	0.918	0.451	0.347
**Corumba, Brazil**	(Galati et al., 1997)	1.524	8	0.733	1.561	0.253
**Colorida, Brazil**	(Galati et al., 2010b)	1.891	8	0.909	1.011	0.311
**Minotauro, Brazil**	(Galati et al., 2010b)	1.433	5	0.890	1.099	0.332
**Barra bonita, Brazil**	(Galati et al., 2010)	0.849	3	0.773	0.637	0.212
**Santana, Brazil**	(Galati et al., 2010b)	2.053	11	0.856	1.561	0.780
**Couto, Brazil**	(Galati et al., 2010b)	1.640	10	0.712	1.242	0.414
**Morro Preto, Brazil**	(Galati et al., 2010b)	1.623	17	0.573	0.451	0.665
**Moeda Sul, Brazil**	(Campos et al., 2020)	1.091	4	0.787	1.011	0.337
**Rola Moça 39, Brazil**	(Campos et al., 2020)	0.786	5	0.488	0.451	0.451
**Rola Moça 40, Brazil**	(Campos et al., 2020)	0.499	3	0.454	0.451	0.000
**Lassance, Brazil**	(Carvalho et al., 2013)	1.886	21	0.620	1.561	0.443
**Pitoco, Brazil**	(de Almeida et al., 2019)	2.169	26	0.666	1.561	0.443
**Parauapebas, Brazil**	(Teodoro et al. 2021)	1.243	16	0.449	1.561	0.665
**Curionóplis, Brazil**	(Teodoro et al. 2021)	1.606	12	0.646	1.099	0.253
*Pains, Brazil* ^&^	(Campos et al., 2017)	1.686	24	0.531	1.330	0.113

**Table 3:** Linear models show no evidence of a latitudinal gradient of species diversity for cave sandflies.

Diversity index	Estimate	Standard Error	t-value	P-value
H	3.099 × 10^−3^	0.016	0.193	0.848
Species number	−0.185	0.155	−1.196	0.241
Evenness	0.011	6.215 × 10^−3^	1.774	0.086
H (rarefied)	6.672 × 10^−3^	0.013	0.500	0.620
Evenness (rarefied)	9.322 × 10^−4^	5.13 × 10^−3^	0.182	0.857
